# Egg-laying increases body temperature to an annual maximum in a wild bird

**DOI:** 10.1038/s41598-022-05516-0

**Published:** 2022-01-31

**Authors:** Magella Guillemette, David Pelletier

**Affiliations:** 1 Département de Biologie, Université du Québec à Rimouski, 300, Allée des Ursulines, Rimouski, QC G5L 3A1 Canada; 2 Département de Biologie, Cégep de Rimouski, 60, Rue de L’Évêché Ouest, Rimouski, QC G5L 4H6 Canada

**Keywords:** Ecophysiology, Metabolism

## Abstract

Most birds, unlike reptiles, lay eggs successively to form a full clutch. During egg-laying, birds are highly secretive and prone to disturbance and predation. Using multisensor data loggers, we show that average daily body temperature during egg-laying is significantly increased (1 °C) in wild eider ducks (*Somateria mollissima*). Strikingly, this increase corresponds to the annual maximum body temperature (40.7 °C), representing a severe annual thermogenic challenge. This egg-laying-induced rise in body temperature may prove to be a common feature of wild birds and could be caused by habitat-related thermoregulatory adjustments and hormonal modulation of reproduction. We conclude our findings with new perspectives of the benefits of high body temperature associated with egg-laying of birds and the potential effect of heat stress that may occur with the future advent of heatwaves.

## Introduction

Laying eggs is a sensitive period for any bird species, as the female produces eggs over a few days to produce a full clutch. During this period, females must ingest sufficient amounts of food and nutrients to fulfil the energetic demand of egg production^1–3^, while various hormones dictate follicular growth and the occurrence of ovulation^4–6^, together with the necessity for any new egg to be fertilised by males during courtship activities. Studies on the phenology of reproduction and laying dates of birds are becoming one of the hallmarks of biological research to understand the effects of climate change on bird populations^7–10^. However, the physiological mechanism by which ambient temperature influences the phenology of egg-laying in birds is still unknown^11^.

The thermal physiology of egg-laying in birds has chiefly been described in species raised for farm production, and there is no information on wild birds. In chickens, quails and turkeys, it is known that laying eggs (oviposition) and ovulation are tightly synchronised and associated with a transient peak in body temperature^12–15^. On the other hand, studies of wild birds have largely been devoted to the estimation of the overall energetic cost of producing a full clutch. The few that have measured metabolic rate of laying birds, using respirometry, have found a slight increase (20–30%) of resting metabolic rate^16–18^. Such an increase in metabolic rate is likely accompanied by an increase in body temperature during egg-laying.

The physiology of wild birds while laying eggs is notoriously difficult to study as they tend to stop laying during or after the experimental measurements^16–18^, while various species will desert their nest if handled or disturbed during this period^19^. Here we present unprecedented data for a wild bird, a large sea duck (*Somateria m. mollissima*) coming ashore to breed every spring to lay 3–6 eggs successively to form a full clutch. We circumvented the methodological hurdle by using year-round monitoring of heart rate (HR) and body temperature (Tb) using data-loggers implanted in the body cavity of 12 breeding females for one year and removed them a year later during subsequent incubation (after laying). These measurements were complemented with data from nest temperature thermistors and regular visual monitoring of activity during the laying process at the breeding colony. We show that egg-laying is associated with a substantial increase in average daily body temperature, which corresponds to an annual maximum. Laying eggs in a wild bird maximises body temperature and might be a common physiological avian feature. We therefore postulated the proximate causes of the observed rise in Tb to be related to thermoregulatory adjustments and hormonal modulation in this species and offer a novel hypothesis stipulating why such a phenomenon would occur from an evolutionary perspective.

### Materials and methods

Study Site, Model Species and definitions. This study was performed on Christiansø Island (55°19'N, 15°12'E), an old Danish fortress located in the southern Baltic Sea, 18 km from the Danish island of Bornholm. About 120 people live on the island along with a colony of about 2600 breeding pairs of Common Eiders^20^. One of the reasons we chose this colony is that habituation of the birds to human presence has developed over the years, facilitating monitoring at the colony, handling and re-capturing the experimental birds. Our study required daily visits to the colony to monitor clutch size and laying phenology, the installation of temperature thermistors at the nests and the implantation of data loggers into incubating females. We obtained a license from Dyreforsøgtilsynet (Royal Veterinarian Corporation) in Denmark, where the data loggers were deployed. All birds were cared for in accordance with the principles and guidelines of the Canadian Council on Animal Care (# CPA 16-03-07-01).

Female Common Eiders *Somateria mollissima* are large sea ducks (1.8–2.8 kg) and lose about 35% of their post-laying body mass by fasting during incubation^21^. Pre-laying females are hyperphagic^22,23^and accumulate fat and protein reserves near the breeding colony and increase their body mass by 32% over winter levels prior to reproduction^24^. The daily time spent diving (DTSD) during the pre-laying period averaged 159.6 min compared to an annual average of 91.4 min^25^. Diving decreased to 70 min during laying and became almost negligible at the onset of incubation.

We define egg laying as the time interval that spans from laying the first to the last egg. We also used the term oviposition as the act of expulsing the egg from the cloaca. Pre-laying and post-laying define the time interval from 0 to 10 days before and after the laying period, the former being associated with rapid follicular growth (see below).

### Reproductive success and breeding phenology

We established a study plot of about 0.5 ha containing 80 nests on the island. We visited the study plot every day, between 16 h 00 and 17 h 00, and 1 April to 31 May from 2003 to 2006 to determine laying dates and incubation periods of females. Our visits did not cause the incubating females to leave their nests unattended. The first and second egg is very often left unattended in this species^21^, and the beginning of laying was defined as the day when the first egg was laid. The total number of eggs laid was determined at the beginning of the incubation period when the female was caught for weighing, banding or band reading. The laying period was defined by clutch size, assuming that one egg was laid each day^26^. This assumption was verified for some females by putting our hands below the attending female to count the number of eggs laid.

### Capture and data logger implantation

We surgically implanted data loggers (DLs) in the body cavity of experimental females (see^27^ for details). All nests and breeding females were identified before implanting the data loggers. In line with previous surgical procedures in this species^27^, we predicted a recovery period of 2–3 days post-implantation to be sufficient and chose experimental females for implantation of data loggers past the 23rd day of their incubation period (incubation time in this population is 26–27 days ^28^). The monitored females were approached slowly and immobilised by putting a black hood over their head, making sure the females did not leave the nest. Then, we removed the experimental females from their nest for implantation. All surgical procedures were conducted indoors, 100 m from the experimental plot. The 45 DLs were 36 mm long (± *SD* = 0.5) × 28 mm (0.2) wide × 11 mm thick (0.3) and weighed 21 g (0.3), which is 1.2% of body mass at implantation^29^. Thirty-nine (87%) experimental females returned to the study area 1 year later, similar to the previously reported survival rate in this species^30^. The last result is most likely related to the fact that implanted DLs do not alter the aerodynamic and hydrodynamic properties of experimental individuals^27^. One year after the implantation, 36 females were re-captured. Only 12 individuals had data logged for a full year, including information about laying behavior and incubation (except for one, for which the logger stopped after laying the first egg). These twelve data loggers recorded (hydrostatic) pressure and heart rate every 2 s and body temperature every 16 s.

### Ambient temperature and nest attendance

Within the same breeding colony, unattended nests with one or two eggs were fitted with a temperature-recording device made up of a dummy resin egg with a temperature sensor embedded on top. This artificial egg was placed in the centre of the nests and fixed to their base with two metal pins. Thermistors were linked to a data logger (Hobo Four-channel External) through a one-meter wire (Onset Computer Corporation, Pocasset, Massachusetts). Loggers were kept in a waterproof casing and buried in the ground to avoid detection by the birds. We programmed loggers to register nest and ambient temperatures every 3 min. Thirteen nest loggers were deployed at the colony in 2004–2006 and recorded data until eggs hatched, after which we removed the device from the nest. From temperature patterns, we extracted the presence at the nest and recess periods of females according to the method described by Sabourin^31^. Recesses lasted from the first time the temperature dropped to the first time the temperature rose. The presence of females at the nest was identified by a decrease or an increase of at least 2 °C within 3 min.We only accounted for recesses longer than 6 min because (1) we considered shorter interruptions were likely representative of on-nest movements and (2) due to the limitation of the sampling frequency. Experimental disturbances of incubating eiders have shown this time interval to be long sufficient to properly record recesses^31^. Moreover, lab experiments with natural nests, aiming at testing the effect of down cover on ambient temperature, describe drops in Tb from 38 °C to 20 °C. These experiments also showed that comparison of nests with and without down result in a difference in the cooling rate of only 0.4 °C over 6 min (0.07 °C/min)^31^. The ambient thermistor was located about 1 m away from the nest and synchronised with the nest thermistor data. We thus computed the ambient air temperature when the female was on and off its nest to detect if the female had any preference for air temperature when leaving its nest. Sea surface temperature (SST) data were obtained for our location for spatial squares of 50 × 50 km from the SST50 database of the NOAA (https://www.avl.class.noaa.gov/glossary/SST50.htm). The SST50 is defined as the daily mean sea surface temperature in degrees Celsius at 1 m depth.

Nest attendance was quantified as the sum of the time spent at the nest for each female using the nest thermistor data. However, egg dumping and nest parasitism are common in eiders (reviewed by Waltho and Coulson^32^), which could bias our estimate of nest attendance during the laying period. In addition, only 2 implanted females (out of 12) were associated with nest thermistors (out of 13). We thus used a second method (called the behavioural method) based on body temperature variation and diving patterns to quantify the time at the nest for the 12 implanted females. Female eiders spend a considerable time foraging before and at the beginning of laying^25^. Body temperature rose, and variation decreased upon arrival at the colony, approximatively at the same time as diving behaviour ceased (see Fig. S1). We used this information to determine the time spent at the nest and time spent on the water. More specifically, for each day and female, we simply subtracted the time spent feeding plus the time spent “resting” between two feeding bouts from 1440 min (24 h) to estimate the time spent at the nest. Resting bouts, that occur between feeding bouts, are considered an obligatory component of foraging in this species as they digest food while swimming and preening on the water^33^. From visual observations, resting bouts following feeding bouts lasted on average 14.9 ± (SD) 14.2 min (range 3.3–64.7 min, n = 52). When resting bouts were longer than one hour between feeding bouts, these events were considered to be spent at the colony. For two nests, we matched data from temperature thermistors with those of the implanted loggers (Fig S1). Results of the two (thermistor and behavioural) methods were highly correlated (R^2^ = 0.881) with a slope of 1.2, for the two implanted females associated with nest thermistors.

### Data analysis and statistics

First, we quantified thermogenesis during egg-laying by calculating daily body temperature (Tb_daily_) for each day of laying as the average of all Tb data (5400 per day) of each individual. A similar approach was used for heart rate with the difference that resting heart rate (RHR) was used as a proxy for resting metabolic rate and computed from a 5 min running average for which the minimum value was extracted for that day and specific bird^29^. These quantities were then compared with RHR during pre and post laying, defined respectively as five days before laying the first egg and five days after laying the last egg. We used five-day periods since the most common clutch size was composed of five eggs, representing 5 days of laying. Once averaged over five-day periods for each bird, the laying period was compared to the pre and post laying periods at the intra-individual level in a before-after fashion using subtraction, giving two deltas per individual. These deltas were averaged across the 12 females, for which confidence intervals (CI) were computed using the bootstrap method. When the **(**95%**)** CI of the average delta was excluding zero, the comparison was declared significant.

Confidence intervals (CI) of means and deltas were performed using R version 4.0.3 (R Core Team, 2020) with *boot* package^34^. CIs were calculated from 10 000 nonparametric bootstrap replicates. The 95% bias-corrected and accelerated method (BCa) intervals are reported. The BCa intervals are the most recommended of the main types of bootstrap confidence intervals^35^. Because many of the CI resulting from the bootstrap method were asymmetric, we reported lower and upper values for each average value.

## Results

### Tb and egg-laying

**Figure 1:**
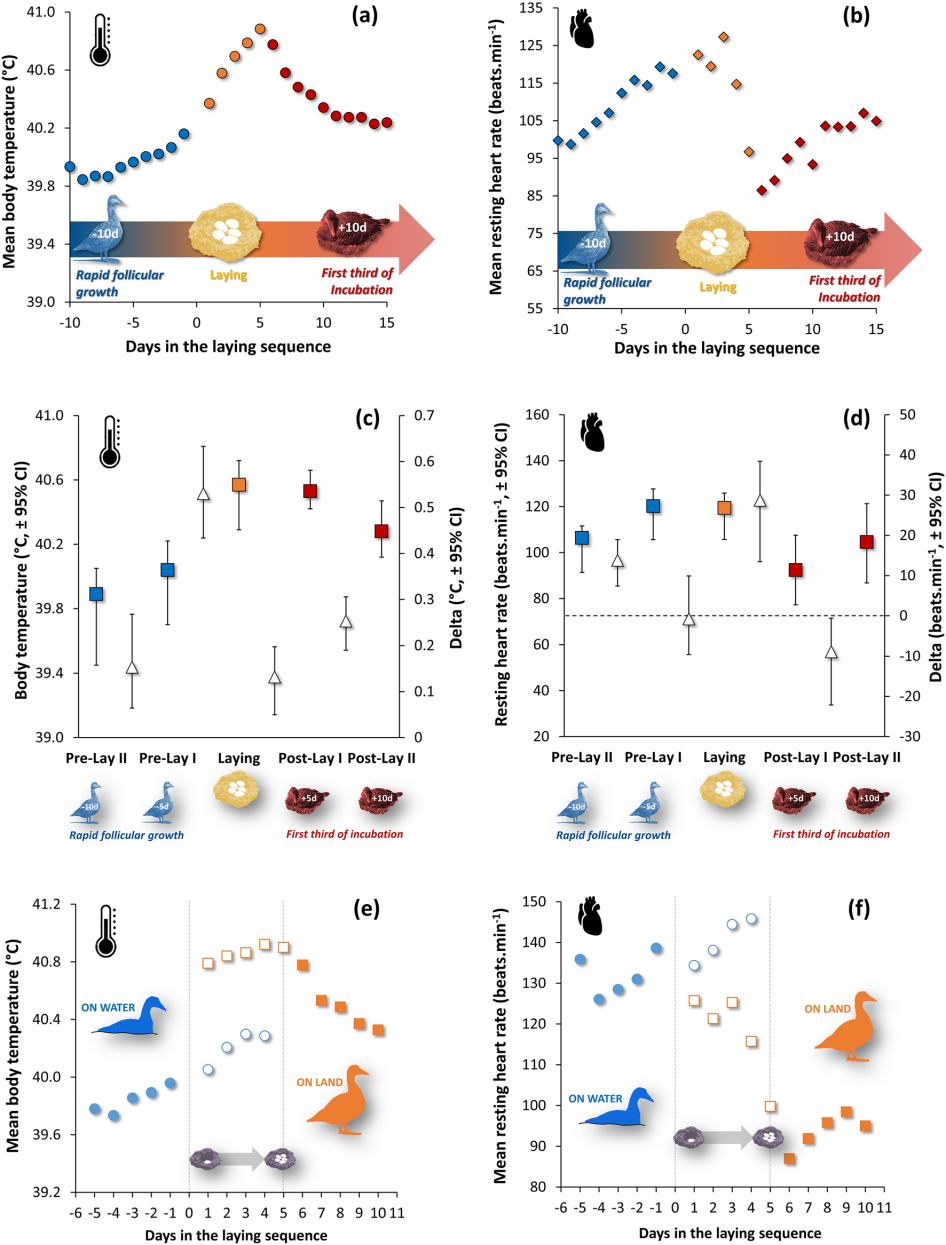
Daily variation of average (24 h) body temperature (**a**) and heart rate (**b**) in relation to the laying sequence of 12 female Common Eiders. The time series shown starts 10 days before (rapid follicular growth) laying of the first egg and ends 10 days after (first third of incubation) laying the last (5th) egg. Each biological stage is shown in a different color and subdivided further on a five-day basis (pre-lay I and II, post-lay I and II), where the average difference between biological stages (triangle) is shown for each period (**c** and **d**). When the bootstrap confidence intervals of the average difference exclude zero, we concluded that a significant difference (5% level) exists between the two time periods. Panels **e**) and **f**) show body temperature and resting heart rate variation relative to the female’s presence on land and water, respectively (see Methods). When exclusively on water, body temperature (spearman r_s_ = 0.967) and RHR (r_s_ = 0.767) are positively related (*p* < 0.05) to days in the laying sequence. In contrast, when exclusively on land, body temperature (r_s_ =− 0.770) and RHR (r_s_ = − 0.781) are negatively related (*p* < 0.05) to days in the laying sequence.

Daily body temperature (Tb_daily_) of laying female eiders varied, on average, by 1 °C for the twelve females during a 25 day period, covering rapid follicular growth (= pre-laying), laying and post-laying (= beginning of incubation). Tb_daily_ was 39.9 °C, ten days before the first egg was laid, peaked at 40.9 °C while laying the fifth egg and decreased to 40.3 °C ten days after the last egg was laid (post-laying period, Fig. 1a). Comparing the laying period to the five days before (Pre-Lay I) and to the five days after laying (Post-Lay I) in a before-after approach (Fig. 1c), Tb_daily_ is significantly higher during laying compared to the pre-laying1 (average delta = 0.53 (CI: 0.43 & 0.63) °C) and post-laying periods (average delta = 0.12 (CI: 0.05 & 0.20) °C).

### HR and egg-laying

Like Tb_daily_, resting heart rate (RHR) steeply increased during the pre-laying period and peaked once the third egg was laid (Fig. 1b). A major difference is that RHR fell precipitously after the fourth egg was laid and while Tb_daily_ was still rising. This fall in RHR continued for the two first days post-laying, only to increase again and to reach a plateau at about 105 bpm between days 6 and 10. As a result, when average values are used over 25 days there is no correlation between mean RHR and mean Tb_daily_ (Pearson’s r = 0.117, n = 25, *p* > 0.05). This result holds true when an intra-individual correlation is calculated for each female separately and then averaged over the 12 females (average r_pearson_ = 0.030 ± (SD) 0.030, n = 12, *p* > 0.05). When laying (116.2 bpm) is compared to the Pre-Lay I period (115.9 bpm) and Post-Lay I (89.6 bpm) periods in a before-after approach (Fig. 1d), RHR is not significantly higher than Pre-Lay I (average delta = 0.3 (CI − 9.6 & 9.9) bpm) whereas the Post-Lay I period is significantly lower (average delta = 28.1 (CI: 13.5 & 38.4) bpm).

### Proximate causes of Tb variations

There are two possible causes for the observed Tb_daily_ variations. The first potential cause is the passage from an aquatic environment to a terrestrial one. Fig. S2a summarises the ambient temperature a female eider experiences when moving from water (4.67 °C) to land (6.21 °C). We know from the work by Jenssen et al.^36^ that the lower critical temperature (LCT) of this species is higher in water at 15 °C (LCT_water_) than in air at 0 °C (LCT_air_). Using the equations provided by Jenssen et al. predicting resting metabolic rate for these two habitats, we conclude that a laying female (2.64 kg) spends 1147 kJ day^−1^ in the water compared to 839 kJ day^−1^ on land, representing a relative difference of 28% in thermogenic requirements. This estimate is corroborated by our measurements of RHR while laying eggs, being on average 145.8 (CI 131.7–161.1) bpm in the water and 117.5 (CI 104.8–126.8) bpm in air, yielding an average and significant difference of − 28.3 (CI − 38.3 & − 19.4) bpm, or 19%. These observations and calculations led us to conclude that the passage from water to land to lay eggs was associated with energy savings, allowing an up-regulation of Tb (see “Discussion”). This, in turn, could explain the increase of Tb while laying eggs, as the time spent on land incubating increases with the laying sequence (Fig. S2b).

However, this mechanism cannot explain all the variation observed in daily Tb during laying. Female eiders spend 95% of their time on land when laying the fifth egg, which increases to a constant value of about 99% after the 5 days of incubation. Thus, from the end of the laying period to about ten days of incubation, Tb_daily_ drops by 0.6 °C, indicating that another factor contributes to Tb variation while laying eggs (Fig. 1a). During the laying period, females are moving back and forth between terrestrial and aquatic environments. Quantifying Tb separately while on land vs Tb while on water for the twelve instrumented females (see Methods and Fig. S1), we show that when individuals were solely on water, body temperature increases significantly (spearman r_s_ = 0.967, *p* < 0.05) from day -5 of the pre-laying period to the end of the laying period by about 0.5 °C (Fig. 1e). A similar observation was made for females exclusively on land, where body temperature decreases significantly (spearman r_s_ = − 0.770, *p* < 0.05) by 0.6 °C (Fig. 1e) from the end of laying to the fifth day of post-laying. Therefore, our method confirms that another factor is causing the observed increase in Tb while laying eggs.

Based on various studies conducted on domestic fowl (see “Discussion”), we hypothesised that a part of the body temperature variation observed during the laying period is due to the ovulation or the very process of ejecting an egg (= oviposition). Ovulation is associated with a transient increase of Tb in domestic fowl occurring 30–90 min after oviposition. One feature of this system is that ovulation occurs a little bit later every day. In this study, we measured the timing of Tb peak during egg-laying of the twelve females and found that it ranged from 41.3 to 41.6 °C (Fig.S2c), lasting between 18 and 30 min. Moreover, peaks in Tb happened significantly later during the laying sequence, although the difference is not significant at the end of this process (Fig. S2c).

**Figure 2:**
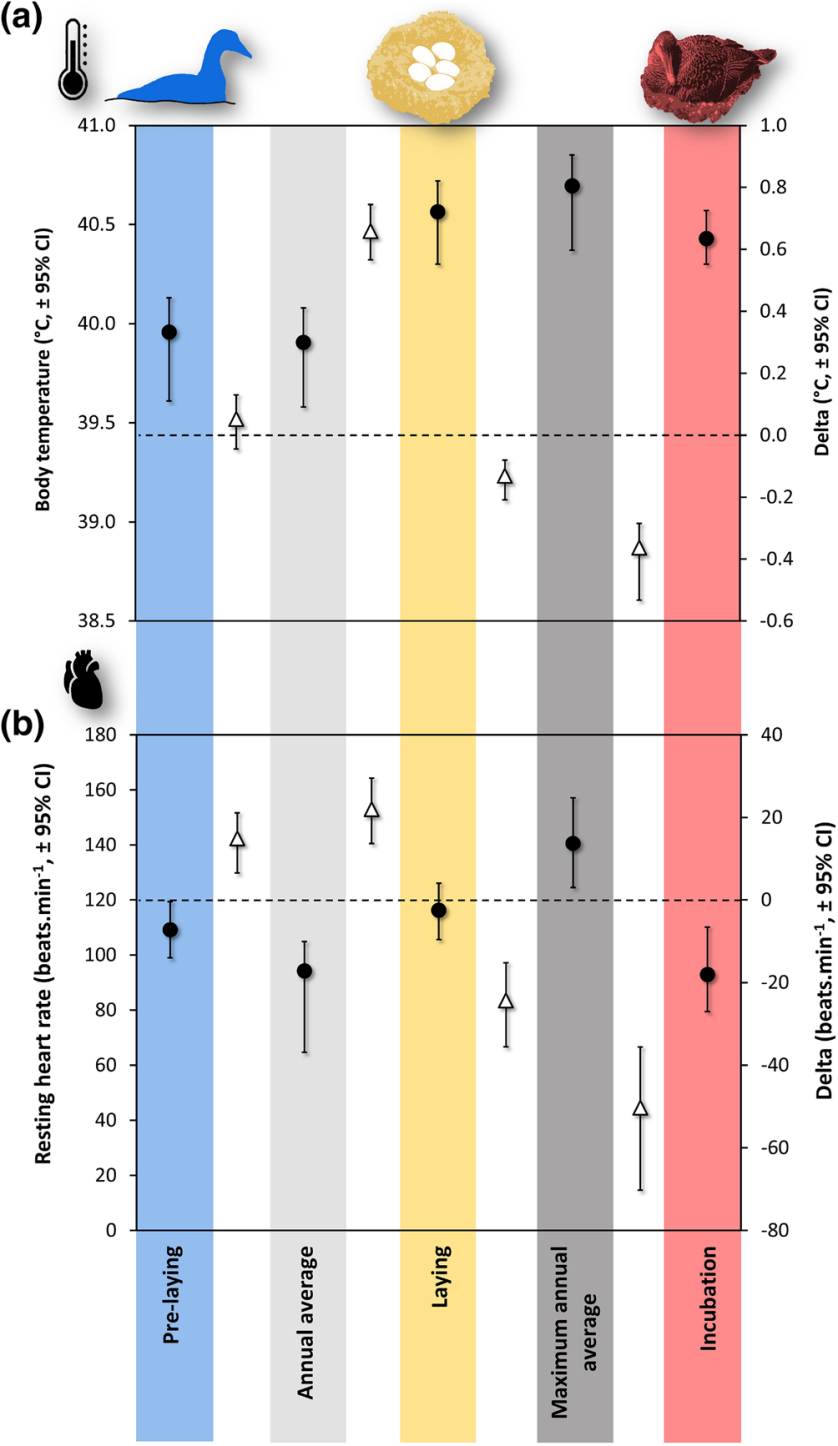
Average daily body temperature (Tb_daily_) and resting heart rate (RHR) during pre-laying (blue, n = 12), laying (yellow, n = 12) and incubation (red, n = 11) of female Common Eiders compared to the annual average (pale grey, n = 12) and the annual maximum running average (dark grey, n = 12). When the bootstrap confidence intervals (95%) of the average difference (triangles) between two adjacent variables exclude zero, it was concluded that a significant difference exists between the two variables compared. Note that the five-day annual maximum running average of Tb_daily_ occurred during laying in 8 females out of 12, whereas none occurred during laying for RHR.

One additional strength of our study is that we can compare the observed body temperature during laying with the full annual cycle (Fig. 2) to gauge the magnitude of this phenomenon. Using a five-day running average of Tb_daily_, for each individual, the average annual maximum Tb_daily_ is 40.70 °C (CI: 0.24), which is 0.13 °C (CI: 0.07) higher than the standard average computed for the laying period. However, this annual maximum running average occurred during egg-laying in 8 females out of the 12, underlining that the egg-laying period contributes largely to the annual maximum observed.

## Discussion

Fifty years ago, the process of laying eggs and ovulation had been associated with an increase in body temperature in domestic fowl^12,13,15,37–40^;. In contrast, data on the thermophysiology of egg-laying in wild birds is entirely lacking. Given that birds are highly secretive, easily disturbed and prone to predation while laying eggs, it may explain the absence of data for wild birds. This is especially true if we consider that a rise in body temperature (Tb) after ovulation and during the luteal phase occurs in various species of domestic (reviewed by^41^) and wild (marsupials:^42,43^; chimpanzees^44–46^:) mammals, including humans (see^47^ for review). In the present study, we report data for heart rate and Tb before, during and after egg-laying of undisturbed wild birds. Not only do we show that egg-laying induces a rise in daily body temperature similar to other vertebrate species, but we also find that daily Tb (Tb_daily_) corresponds to an annual maximum, representing an annual thermogenic challenge for these birds. Here we propose that habitat-related thermoregulatory adjustments and hormonal modulation of reproductive activities cause the observed peak of thermogenesis during laying and then discuss the implications of our findings.

### The energetic cost of egg-laying

Resting metabolism increases during reproduction in various vertebrates, including placental and marsupial mammals, reptiles and birds^48,49^. In this paper, we have shown that resting heart rate (RHR), often used as a proxy for resting metabolic rate^50,51^, increases steadily during the pre-laying and laying period in eiders (until the fourth egg is laid), in concert with Tb_daily_. We interpret such an increase in RHR due to the growth of new tissues, such as the oviduct and ovary, which add to the laying hen's metabolic activity (productive costs). Birds are known to build up reproductive organs rapidly, and from pre-laying eiders dissected at various stages^22,52,53^, it was estimated that the ovary and oviduct increases by a factor of 5 in two weeks, increasing whole body mass by 7%. In addition, it has been estimated that eiders require about 6 days to fully develop a follicle^3^, a period called rapid follicular growth (RFG), and about ten days to produce a clutch of five eggs. The observation that RHR fell precipitously after the fourth egg was laid supports the notion of an increased energetic cost during the growth of the oviduct and preovulatory follicles, while RFG should be completed at this stage (Fig. 2b). However, there is a competing interpretation for the reduction in RHR towards the end of egg-laying; it may be caused by the increased time spent on land and the associated reduction in thermoregulatory costs (see below). Clearly, the isolation of the key mechanism behind the energetics of egg-laying in wild, laying birds is highly complex (even in an experimental setting, see^16,18^). There is a diverse array of factors that may influence resting metabolism, such as the thermogenic effects of hormones, body mass fluctuations while laying^28^, behavioural and physiological compensation^54^ and, for aquatic birds, habitat-related variation in thermoregulatory costs^36^. Only an experimental approach and a detailed quantification of all these variables would lead to a full understanding of energy allocation during egg-laying in birds, which is outside of the scope of this paper.

### Coming ashore to lay eggs: thermoregulatory adjustments

During the breeding season, any marine bird species has to move from an aquatic to a terrestrial environment where heat conduction in the air is reduced by a factor of 20–24 compared to water. Thus, moving temporarily from an aquatic medium to a terrestrial one requires thermoregulatory adjustments. We observed that Tb in laying female eider ducks is 0.6 °C higher on land than when on water. This rise in Tb is similar to former studies monitoring Tb of birds while moving from the water to land^55–59^ (but see^60^), but also of semi-aquatic mammals like muskrats^61^, beavers^62^ and seals^57^. We also observed that RHR decreased by 19% during laying when moving from water to land. Because RHR in female eiders falls when leaving water at the same time as Tb rises, we conclude that female eiders are saving energy due to the conductive/insulative advantages, allowing the regulation of Tb to a higher level. However, once the last egg is laid and most of the time (95%) is spent on land, Tb_daily_ drops by 0.5 °C, indicating that, in addition to the habitat shift described above, another factor is playing a vital role in the observed Tb variation.

### Hormonal modulation of egg-laying: effect of progesterone?

We consider the hormonal cascade associated with reproduction a key factor causing the observed rise in Tb of female eiders. It has been shown experimentally in humans^63^ and domestic mammals^41^ that a rise in Tb is caused by a progesterone surge during the luteal phase after ovulation, although such evidence is lacking in birds^14^. Ovulation in birds, as in mammals, is hormonally controlled by the hypothalamus–pituitary–gonadal axis (reviewed by^64,65^). The preovulatory surge of progesterone (PG) and luteinising hormone (LH) occurs 4–6 h before ovulation with a positive feedback mechanism in domestic birds^6,66,67^, whereas the secretion of PG and LH seems to occur coincidently in wild ducks^68,69^. At the time scale of a full clutch, the progesterone level of wild mallards in captivity, canvasbacks^68,69^ and canaries^70^ is much higher during laying when compared to the pre and post-laying periods. A similar observation was made for domestic hens in which progesterone decreased markedly during laying pauses^71,72^. Because progesterone is associated with thermogenic effects^73,74^, we hypothesise PG to be partly responsible (together with the habitat-related shift described above) for the high Tb_daily_ observed during laying. However, prostaglandin (PGF_2_ and PGE_2_), another hormone that causes a thermogenic effect^75,76^, can be produced in various tissues, including preovulatory follicles. Prostaglandins are responsible for egg movement under oviduct contractions and oviposition (ejection) of the new egg in domestic fowl^77–79^. In addition, thyroid hormones (THs) are also known to vary between reproductive and non-reproductive states of birds and is also associated with thermogenic effects^80–82^. For example, it has been hypothesised that triiodothyronine (T3) secretion triggers photosensitivity by increasing in the medial basal hypothalamus (MBH) under long photoperiods and interacting with gonadotropin-releasing hormone (GnRH) production^81^. However, such a hypothesised effect would occur a long time before egg-laying. Another effect of THs is that it would trigger photorefractoriness under the influence of long days, thus putting an end to reproduction^64,80^. Gabrielsen et al.^83^ have measured the level of T3 in breeding Common Eiders, which show a low level of T3 during the laying and pre-laying state, which increases regularly then during the incubation period. This suggests that T3 is unlikely to be the hormone that triggers a rise in body temperature while laying eggs in this species, although further examinations are warranted.

In support of the hormonal modulation hypothesis of Tb, we observed a transient peak of Tb in female eiders, when on land and while laying eggs (Fig S1d, Fig S2 cd), as observed for chickens^12,13,39^ turkeys^14^ and quails^15^, lasting 22 min on average. One peculiar aspect of the hormonal control of egg-laying is its circadian dependency, where a subsequent LH surge must occur slightly later than 24 h after the previous surge (the open window model, see^6^). Given that ovulation occurs 15–90 min after oviposition and both processes are tightly synchronized^5^, oviposition commences a little bit later every day, as exemplified in hens^12,13,40^. In the present study, we observed a daily peak of Tb occurring later every day for eggs 1 to 3, but not for the 4th and 5th egg. Based on the high frequency of nest monitoring for female eiders breeding in northern Canada, Watson and colleagues similarly reported that eggs were increasingly delayed during the laying sequence with an average laying interval of 28 h^26^. Altogether, these results support the open window model of egg-laying in common eiders and suggest that the rise of Tb associated with egg-laying in female eiders to be partly hormonal. However, the peak of Tb caused by prostaglandin around oviposition in domestic fowl is transient and would not explain the full magnitude of Tb elevation observed in laying eider ducks. We thus speculate that although both progesterone and prostaglandin can influence Tb_daily_ in female eiders, the effect is predominantly caused by progesterone, given the prevalence of its secretion while laying eggs in wild ducks^68,69^ and canaries^70^.

### Hyperthermia or fever?

A rise in metabolic activity has been widely observed throughout the vertebrate lineage, including placental mammals, marsupials as well as ectotherms like lizards and turtles^48,49^. Not only do some of these vertebrates increase their metabolic rate during reproductive periods, but Tb and thermoregulation are also altered. Based on these observations, the parental care model of endothermy has been proposed^48,49^, suggesting that the high level of metabolic activity associated with breeding in various species of vertebrates was the main incentive for the evolution of a high and stable body temperature. For instance, high incubation temperature accelerates embryonic growth and decreases the duration of incubation in birds^84–86^. The main factors that assure a high incubation temperature for the embryo are nest attentiveness and a high Tb^87^. However, evidence for the latter is rare^88^. Here we show that Tb_daily_ at the beginning of incubation in common eiders was 0.5 °C higher than the annual average. Although such a difference may seem small, recent evidence^84–86^ has shown that differences in incubation temperature of this magnitude can affect various aspects of the phenotypic quality of chicks (locomotor performance, acquired immune responses, thermoregulation abilities, etc.). This is particularly important in incubating female eider ducks that display one of the highest levels of nest attendance during incubation amongst birds (95–99%^21^), leaving variation of Tb as the second most important modulator of incubation temperature.

However, the above interpretation does not resolve the observation of an even higher level of Tb_daily_ during egg-laying in this study. Although a rise in body temperature during ovulation has been described in various vertebrates, we are not aware of any hypothesis explaining this phenomenon from an evolutionary perspective. Here we propose that the observed rise in body temperature during egg-laying of female eiders may be analogous to an infection-induced fever, an up-regulation of Tb as a consequence of a change to the thermoregulatory set-point. Infection-induced fever is a cardinal response to pathogens that has been preserved in endotherms and ectotherms over millions of years of evolution (including humans^89^ and birds^76^). We, therefore, hypothesise that the increased thermogenesis during egg-laying in eiders is an anticipatory response governed by the need to boost the immune system during the fertilisation process. The reproductive tract of birds (oviduct) is prone to infection even if the oviduct and rectum are separated; they both protrude to the outer orifice, the cloaca. Most male and female birds achieve copulation by the bondage of their cloaca, also known as "cloacal kisses". It has been shown in several studies that the cloaca of females and males contains a large diversity of bacteria^90–92^, while manipulative experiments have shown that males transmit bacteria to females during copulation^91^ and that infected females may transmit pathogens to their eggs^93^. Pathogens transmitted through copulation may impair the survival of the future zygote, and the increased likelihood of sexual disease transmission can alter the host's survival^91^. Therefore, a high body temperature during ovulation and the formation of the egg would boost the innate immune system in a manner as described by Evans and colleagues^89^ and reduce the likelihood of any infectious agents surviving within the oviduct.

### Implications: heatwaves, egg quality and laying dates

The impact of environmental temperature on endotherms is often envisioned through the concept of critical thermal limits (CTL), being the lethal Tb boundaries within which an individual is likely to survive^94^. However, at the population level, lower thermal limits have to be considered, as reproduction (and other functions) might be compromised under heat stress before an individual’s survival is jeopardised^94,95^. This is especially true when "natural" thermal boundaries of the annual cycle and reproduction coincide, as shown in female Common eiders in this study. For example, average peak Tb while laying eggs (ranging from 41.3 to 41.6 °C for about 20 min, Fig.S2) compared with intense locomotion during migratory flights (approximately 41.5 °C on average for flights > 80 min^96^), in the same individuals as in this study, is similar. However, migrating eiders limited the development of flying-induced hyperthermia by landing on the water surface regularly to cool down^96^. At the scale of a whole day, the average Tb_daily_ during laying ranged from 40.4 to 40.9 °C (Fig. 2), whereas the average Tb_daily_ during migration days was 40.4 °C. In this context, egg-laying may represent a sensitive period relative to ambient temperature fluctuations that may occur naturally.

Apart from increases in average temperatures, one central prediction of climate change models is that it will also increase temperature variability in the form of heatwaves, which are predicted to increase in frequency and duration in the future^97^. Studies investigating the phenology of egg-laying in birds has attracted considerable attention and are becoming one of the hallmark effects of climate change on animal populations overall. Despite correlative evidence showing a negative relationship between ambient temperature and laying dates^7,9^, there are only limited experimental studies showing that spring temperatures affect the timing of laying directly^8,10^. Interestingly, in the sole study, we are aware of that measured the effect of changes in ambient temperature on laying in wild birds, Schou et al.^98^ identified a critical thermal window in captive ostrich (*Struthio camelus*), for which the rate of egg-laying peaked at 20 °C, dropping by 15% and 18% when temperatures increased and decreased by 5 °C, respectively. However, it is unknown which mechanisms contributed to these results.

Here, we suggest that the inverse relationship between laying dates of birds and ambient temperature^7,9^ is an avoidance strategy preventing additional heat loads while producing eggs. As ambient temperature increases under the effect of global warming, birds would show a tendency to lay earlier. However, such a hypothesis alone would not explain the observation that birds lay eggs later at colder ambient temperatures. In that regard, Stevensen and Bryant^99^ have proposed the possibility that ambient temperature may act as a constraint with colder ambient temperature forcing laying birds to allocate more energy to thermoregulation, thereby reducing any available energy to reproduction. Nevertheless, these findings support the notion of fertility thermal limits, where reproduction and the process of egg-laying might be affected at an ambient temperature much below those identified as critical thermal limits for survival.

## Conclusions

In conclusion, this is the first study demonstrating that egg-laying in a free-ranging wild bird is associated with a substantial increase in Tb. Based on the laboratory model of domestic fowl and the general prevalence of metabolic increases during reproduction in various vertebrates, we suggest that the rise of Tb while laying eggs may be a general phenomenon in birds. We speculate that the mechanism triggering the increased thermogenesis is a regulated process, comparable to fever, in order to upregulate the immune system. Given the panoply of recent studies into the temperature-dependent phenology of egg-laying in birds, we believe the study of the specific physiological effects of ambient temperature on egg-laying birds will be an essential and exciting line of inquiry for the years to come.

## Supplementary Information

Supplementary Information.

## Data Availability

All data are available in the main text or the supplementary materials.
