# Supplementary material for: Egg-laying increases body temperature to an annual maximum in a wild bird
Source: Sci Rep. 2022 Jan 31;12:1681. doi: 10.1038/s41598-022-05516-0 (PMC8803923; doi:10.1038/s41598-022-05516-0)
Supplement: Supplementary file 1 — Supplementary Information. [file 41598_2022_5516_MOESM1_ESM.pdf]

Supplementary Materials for

Egg-laying increases body temperature to the annual maximum in a wild bird

M. Guillemette, David Pelletier.

Correspondence to: magella_guillemette@uqar.ca

This PDF file includes:

Figs. S1 to S2

Fig S1. Physiological and behavioural monitoring of one female eider duck (RW328) during the ovulation and laying process. Example of **A**) variation in body temperature, **B**) the timing of each dive (depth), and **C**) variation in heart rate over a 24 h period. The yellow panel superimposed on the figure corresponds to data from the nest thermistor (see Methods). It shows the approximate time the female was on the nest and indicates that body temperature while on land is less variable. **D**) Variation of body temperature over a 24 h period for female RW328 for five days of ovulation, starting with the day before laying the first egg. On all panels, the time of observation at the nest is shown (binoculars with arrow). The three last panels are superimposed with a yellow rectangle, showing the female's time on the nest as indicated by the nest thermistor. Further, the daily peak in body temperature is displayed on each panel and was used to test the hypothesis (for each female) that such a peak in Tb is delayed each day, as dictated by the hormonal and circadian driven process of ovulation (see Fig. S2**D**).

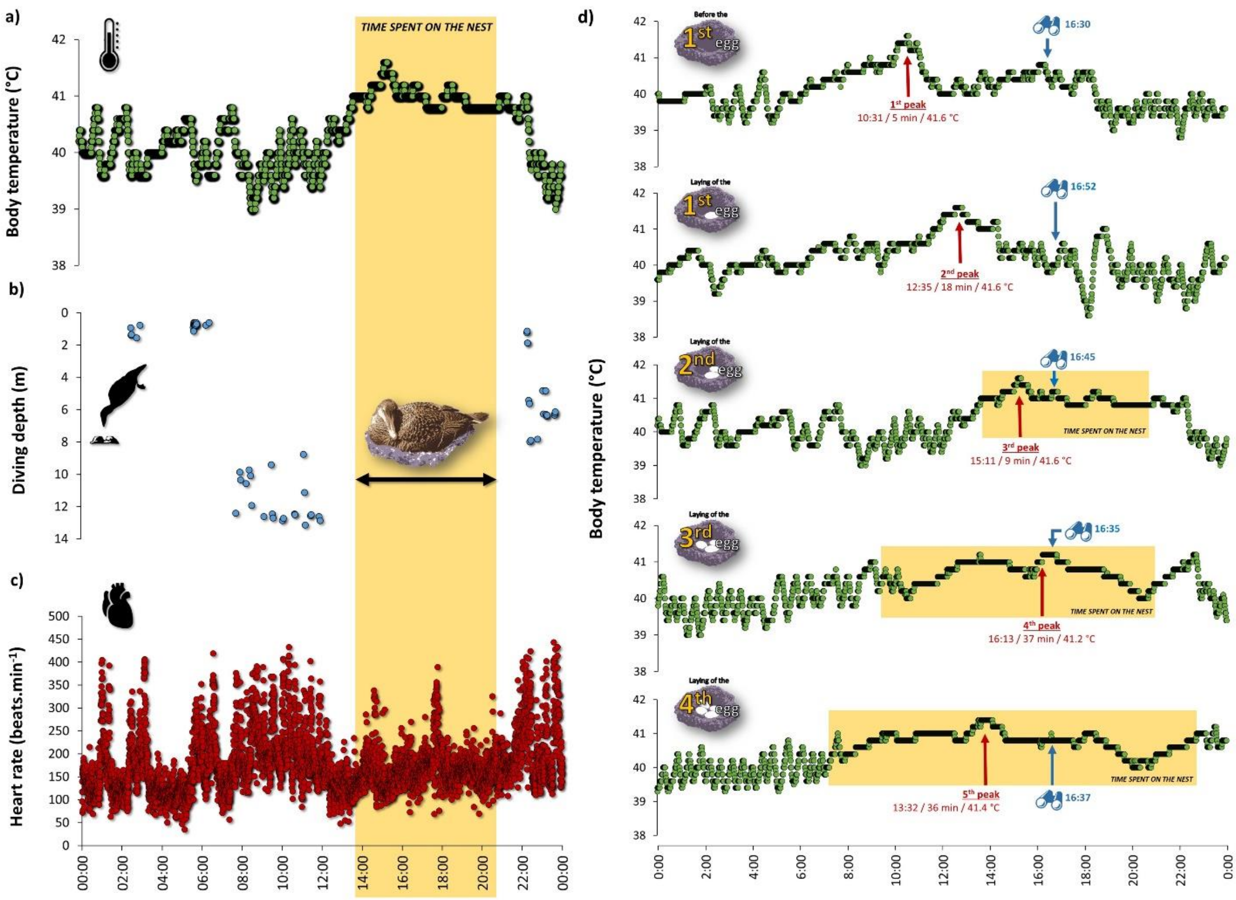
a)
TIME SPENT ON THE NEST
Body temperature (°C)
b)
Diving depth (m)
c)
Heart rate (beats.min-1)
d)
Before the 1st egg
1st peak
10:31 / 5 min / 41.6 °C
16:30
Laying of the 1st egg
2nd peak
12:35 / 18 min / 41.6 °C
16:52
Laying of the 2nd egg
3rd peak
15:11 / 9 min / 41.6 °C
16:45
TIME SPENT ON THE NEST
Laying of the 3rd egg
4th peak
16:13 / 37 min / 41.2 °C
16:35
TIME SPENT ON THE NEST
Laying of the 4th egg
5th peak
13:32 / 36 min / 41.4 °C
16:37
TIME SPENT ON THE NEST
Body temperature (°C)

Figure S2. **Body temperature in eider ducks in dependence of nest attendance and during laying sequence. A)** Ambient air and water temperature when females are on or off the nest (see Methods) together with the lower critical temperature (LCT) for female Common Eiders while on land or in water as determined by Jenssen et al. [20]. **B**) Variation in daily average nest temperature during the laying sequence and the time spent on the nest as quantified by two different methods. The behavioural method data stem from 12 females instrumented with implantable loggers, whereas the thermistor method data were derived from 11 different females plus two implanted females (see Methods). **C**) Average level of Tbmax during laying and incubation and **D**) timing of Tbmax in relation to the laying sequence, testing the hypothesis that Tbmax occurs later every day of the laying sequence compared to incubation.

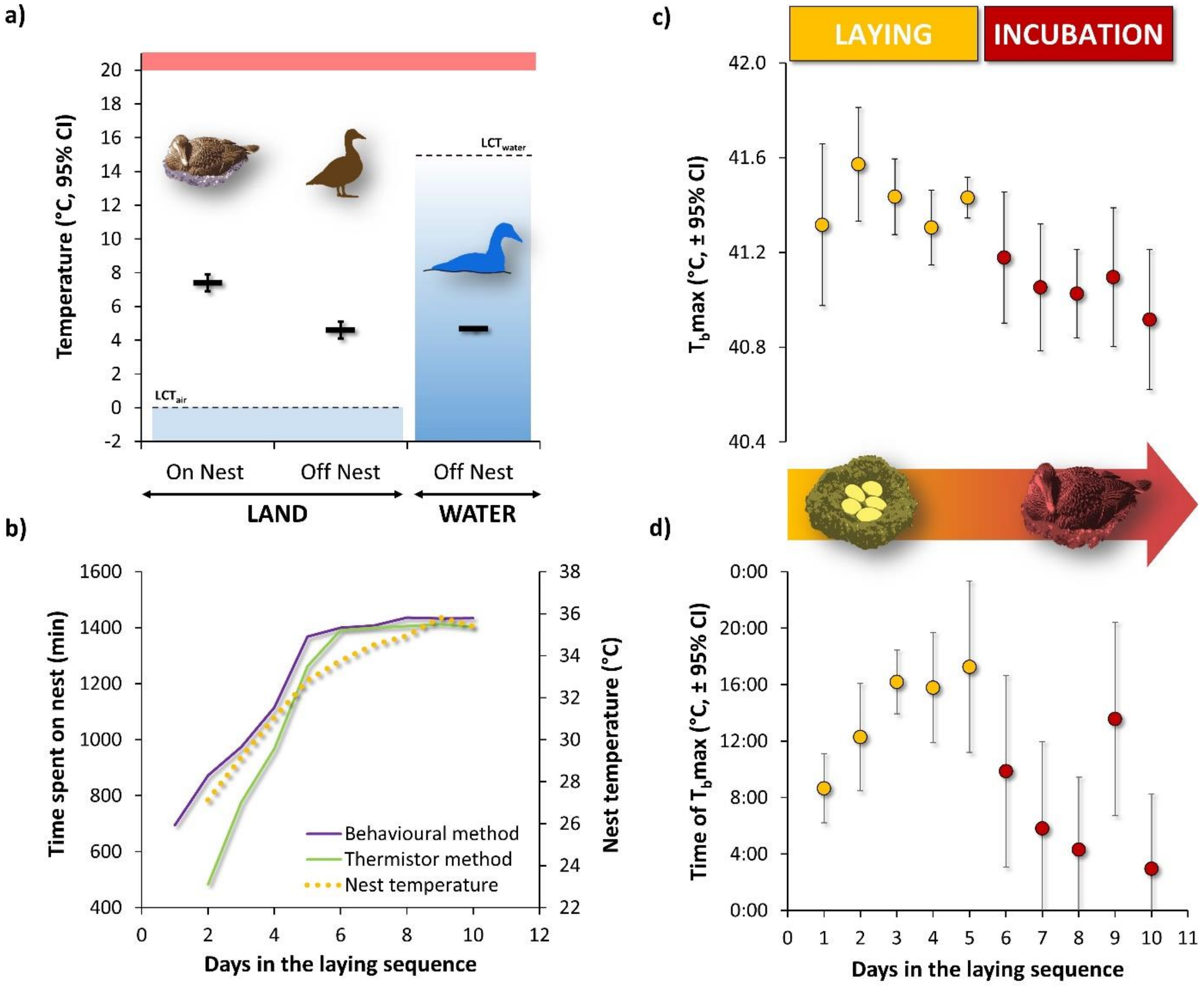
a)
Temperature (°C, 95% CI)
LCT$_{water}$
LCT$_{air}$
On Nest
Off Nest
Off Nest
LAND
WATER
b)
Time spent on nest (min)
Nest temperature (°C)
Behavioural method
Thermistor method
Nest temperature
Days in the laying sequence
c)
LAYING
INCUBATION
$T_b$max (°C, ± 95% CI)
d)
Time of $T_b$max (°C, ± 95% CI)
Days in the laying sequence
